# Inverse changes in L1 retrotransposons between blood and brain in major depressive disorder

**DOI:** 10.1038/srep37530

**Published:** 2016-11-22

**Authors:** Shu Liu, Tingfu Du, Zeyue Liu, Yan Shen, Jianbo Xiu, Qi Xu

**Affiliations:** 1 National Laboratory of Medical Molecular Biology, Institute of Basic Medical Sciences & Neuroscience Center, Chinese Academy of Medical Sciences and Peking Union Medical College, Tsinghua University, Beijing, 10005, China; 2 Institute of Medical Biology, Chinese Academy of Medical Sciences and Peking Union Medical College, Kunming, 650118, China

## Abstract

Long interspersed nuclear element-1 (LINE-1 or L1) is a type of retrotransposons comprising 17% of the human and mouse genome, and has been found to be associated with several types of neurological disorders. Previous post-mortem brain studies reveal increased L1 copy number in the prefrontal cortex from schizophrenia patients. However, whether L1 retrotransposition occurs similarly in major depressive disorder (MDD) is unknown. Here, L1 copy number was measured by quantitative PCR analysis in peripheral blood of MDD patients (n = 105) and healthy controls (n = 105). The results showed that L1 copy number was increased in MDD patients possibly due to its hypomethylation. Furthermore, L1 copy number in peripheral blood and five brain regions (prefrontal cortex, hippocampus, amygdala, nucleus accumbens and paraventricular hypothalamic nucleus) was measured in the chronic unpredictable mild stress (CUMS) model of depression in mice. Intriguingly, increased L1 copy number in blood and the decreased L1 copy number in the prefrontal cortex were observed in stressed mice, while no change was found in other brain regions. Our results suggest that the changes of L1 may be associated with the pathophysiology of MDD, but the biological mechanism behind dysfunction of L1 retrotransposition in MDD remains to be further investigated.

Major depressive disorder (MDD) is a common psychiatric disorder that is defined by episodes of depressed mood lasting for more than 2 weeks with the symptoms of disturbed sleep and appetite, excessive guilt, reduced concentration, slowed movements and suicidal thoughts^1^. MDD affects approximately 17% of the population, and places a heavy burden on the family and society^2^. Previous studies demonstrated that both genetic and environmental factors were involved in the etiology of MDD^1^,^3^. However, it is not yet to clarify how the environmental stressors interact with genetic factors in MDD^1^.

Increasing evidence suggests that mobile elements are regarded as sensors of environmental stress in the brain^4^. Long interspersed nuclear element-1 (LINE-1 or L1), as one of mobile elements, remains active in both human and mouse genome^4^,^5^. L1 is an autonomous element spanning about 6 kb of DNA and encodes a 5′-UTR containing an internal promoter and two open reading frames, ORF1 (an RNA binding protein) and ORF2 (an endonuclease and reverse transcriptase protein)^4^,^5^. Normally, L1 is heavily methylated in the normal state and hypomethylation of the L1 promoter may lead to its retrotransposition, which can change the expression of nearby genes and the genome structure^6^,^7^. Increasing evidence has recently suggested that L1 retrotransposition may occur in somatic cells, such as cancer cells and neural progenitor cells. In addition, L1 retrotransposition can cause the mosaicism in brain tissues. Recent research has revealed that brain tissues seem to have more retrotransposition than non-brain tissues, suggesting an important role of L1 in the central nervous system^8^. Furthermore, studies in patients and animal models have demonstrated that L1 dysregulation may contribute to neurological disorders, such as Rett syndrome and post-traumatic stress disorder (PTSD). Recently, L1 retrotransposition was found to become active in the postmortem prefrontal cortex of patients with schizophrenia and to be involved in depression and bipolar disorder^4^,^9^.

In this study, we quantified L1 copy number in peripheral blood genomic DNA and found that patients with MDD had a significant increase in L1 copy number. We also measured the methylation levels of L1 5′-UTR and found that MDD patients had less methylation levels in peripheral blood than healthy controls. Next, we quantified L1 copy number in the mouse model of depression (chronic unpredictable mild stress, CUMS), and found that CUMS mice had higher L1 copy number in peripheral blood than controls, consistent with results from MDD patients. Chronic unpredictable stress causes major structural and functional maladaptations in multiple brain regions^10^,^11^. In this study, we further determined L1 copy number within 5 different brain regions involved in emotion regulation, including the prefrontal cortex (PFC), hippocampus, amygdala, nucleus accumbens (NAC) and paraventricular hypothalamic nucleus (PVN). Intriguingly, we found that L1 copy number in genomic DNA was different between blood and brain regions, and was decreased in PFC of stressed mice, while there were no significant changes in other brain regions.

## Results

### Increased peripheral blood L1 content in MDD patients

**Figure 1:**
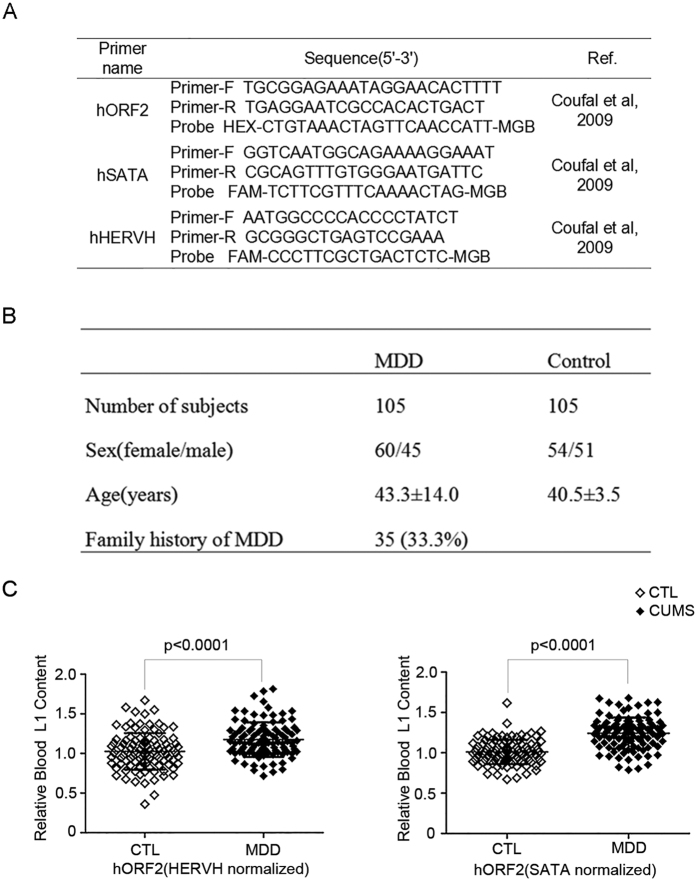
Increased L1 copy number in peripheral blood of MDD patients. **(A**) Primers for the method of Taqman used in human. **(B**) Summary of the demographic variables of blood samples. (**C**) L1 ORF2 copy number was measured with SATA or HERVH as internal controls by comparative Ct methods. Hollow and solid diamonds were used to represent values of control and MDD subjects, respectively. Binary logistic regression was used to determine p values adjusted for age and sex. Data are means with SD. CTL, controls (n = 105); MDD, major depressive disorder (n = 105).

Quantitative multiplex polymerase chain reaction (PCR) to detect L1 copy number in the human brain regions and other tissues was first developed by Coufal *et al*.^12^, which was then widely used to evaluate the L1 copy number in neurological disorders such as Rett syndrome (RTT)^13^ and schizophrenia^9^. We applied the same method to compare L1 copy number in MDD patients and healthy controls in this study. L1 ORF2 copy number in peripheral blood was quantified by quantitative real-time (RT)-PCR with two internal controls, designed for alpha-satellite (SATA) and human endogenous retrovirus (HERVH). The primers and probes used for RT-PCR amplification of L1 are listed in Fig. 1A. The clinical features and demographic information of subjects are presented in Fig. 1B. As shown in Fig. 1C, a significant increase in L1 copy number was observed in MDD patients compared to control subjects (p < 0.0001 adjusted for age and sex in HERVH normalized L1 levels and p < 0.0001 adjusted for age and sex in SATA normalized L1 levels). The age was not correlated with L1 copy number(r = −0.100, p = 0.310 for HERVH normalized L1 levels and r = 0.097, p = 0.361 for SATA normalized L1 levels). The total score of HAMD-17 was not correlated with L1 copy number (r = −0.052, p = 0.603 for HERVH normalized L1 levels and r = 0.108, p = 0.274 for SATA normalized L1 levels).

### Decreased methylation of L1 5′-UTR in MDD patients

**Figure 2:**
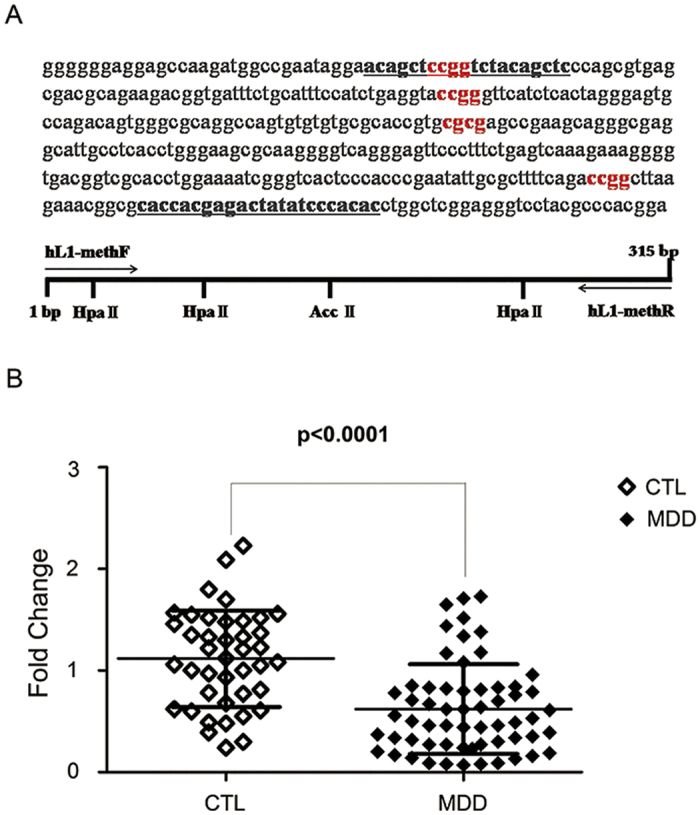
Hypomethylation of L1 5′-UTR in peripheral blood of MDD patients. **(A**) Top: The sequence of L1 5′-UTR chose to measure the methylation level. Bottom: Schematic diagram containing primers and four CpG restriction enzyme sites. **(B)** Significantly lower methylation level in MDD patients. Hollow and solid diamonds were used to represent values of control and MDD subjects, respectively. Binary logistic regression was used to determine p values adjusted for age and sex. Error bars indicate SD. CTL, controls (n = 41); MDD, major depressive disorder (n = 60).

Methylation sensitive restriction enzymes (MSREs) with RT-PCR were used to measure L1 5′UTR DNA methylation. A 315 bp fragment rich in CpG sites was amplified and four CpG restriction enzyme sites that can be cleaved by the methylation sensitive restriction enzymes (HpaII, AccII). The schematic diagram is shown in Fig. 2A. L1 5′UTR exhibited significantly lower methylation level (Total of four CpG sites, −43.9%, p < 0.0001) in peripheral blood of MDD patients than control subjects (Fig. 2B). In addition, the age and total score of HAMD-17 was not correlated with L1 5′-UTR DNA methylation (r = 0.009, p = 0.945; r = −0.215, p = 0.1).

### Decreased sucrose consumption and increased immobility time in CUMS

**Figure 3:**
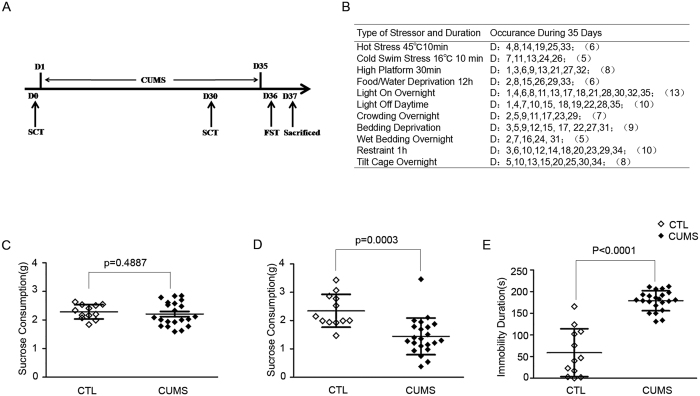
Depressive–like behaviors in mice resulted from chronic unpredictable mild stress (CUMS). **(A**) Schematic diagram of chronic unpredictable mild stress (CUMS) paradigm. (**B**) Daily schedules for the Chronic Unpredictable Mild Stress. (**C**) Sucrose consumption was similar between controls and stressed mice before the CUMS procedure. (**D)** After the CUMS procedure, sucrose consumption was lower in the stressed mice compared with control. (**E)** Immobility time was significantly decreased in the stressed group. The p value was evaluated by Mann-Whitney U test, data are means with SD. CTL, controls (*n* = 12); CUMS, Chronic unpredictable mild stress (*n* = 21).

To further examine the changes of L1 copy number in MDD, we established the depression model of mice via chronic unpredictable mild stress (CUMS) paradigm, which is the widely applied and most accepted animal model of depression. CUMS was firstly reported by Katz and Hersh, and further improved by Willner *et al*.^14^,^15^. The daily schedules are summarized in Fig. 3B. These two phenotypes could be revealed through sucrose consumption test and forced swimming test, respectively. After CUMS, sucrose consumption and forced swimming test were employed to determine the depressive phenotypes. As shown in Fig. 3C, sucrose consumption was similar in the beginning of the CUMS procedure across groups. After 35 days of CUMS, significant decrease in sucrose consumption was observed in the CUMS group, suggesting depressive-like behavior in CUMS mice (*p* = 0.0003, Fig. 3D). In addition, stressed mice displayed significantly increased immobility time and decreased swimming time compared to control mice during forced swim test (FST) after 35-day experiment (*p* < 0.0001, Fig. 3E). The phenotype of increasing immobility time in stressed mice indicated that mice in the CUMS group also displayed depressive phenotype.

### Increased L1 content in blood and decreased L1 content in the prefrontal context in CUMS model

**Figure 4:**
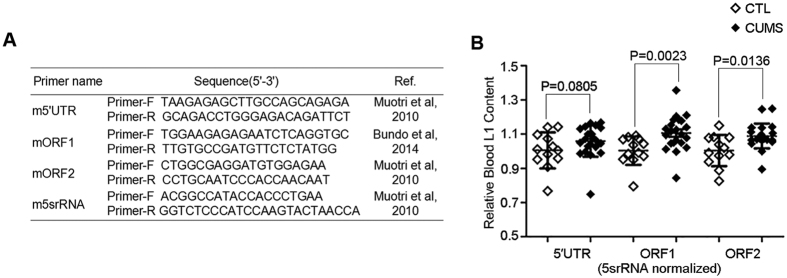
Increased L1 copy number in peripheral blood of CUMS mice. **(A)** Primers for the method SYBR Green used in mouse. (**B)** L1 copy number (5′UTR, ORF1, ORF2) was measured with 5s-rRNA as internal controls by comparative Ct methods. Significantly higher L1 copy number in stressed mice was observed compared with controls (5′UTR, 5.4%, p = 0.0805; ORF1, 10.3% fold, p = 0.0023; ORF2, 8.4%, p = 0.0136). Mann-Whitney U test was used to determine p values. Error bars show SD. CTL, controls (*n* = 12); CUMS, Chronic unpredictable mild stress (*n* = 21).

Muotri *et al*. were the first to develope an approach based on single cell genomic qPCR to detect the activity of endogenous L1 retrotransposition within the mouse genome^13^. Bundo *et al*. quantified brain L1 content in a schizophrenia animal model that showed increased L1 content as well as in schizophrenia patients^9^. With the same method, we tested two questions in mouse models of depression: whether blood L1 change was similar between depression patients and CUMS mice and how L1 is changed in different limbic brain regions of stressed mice? Firstly, we isolated genomic DNA from peripheral blood and quantified L1 copy number by quantitative RT-PCR with 5srRNA as internal control. L1 copy number was detected using different methods in human and mouse samples because the distributions of L1 copy number in the human genome are less frequent than in the mouse genome; Taqman method is more sensitive than others reported to date. The primers for RT-PCR amplification of mouse L1 are presented in Fig. 4A. We observed a significantly elevated L1 copy number (5′UTR, 5.4%, p = 0.0805; ORF1, 10.3%, p = 0.0023; ORF2, 8.4%, p = 0.0136) compared to controls, consistent with results from MDD patients (Fig. 4B). This result suggests that increased L1 content in peripheral blood may be related to MDD.

**Figure 5:**
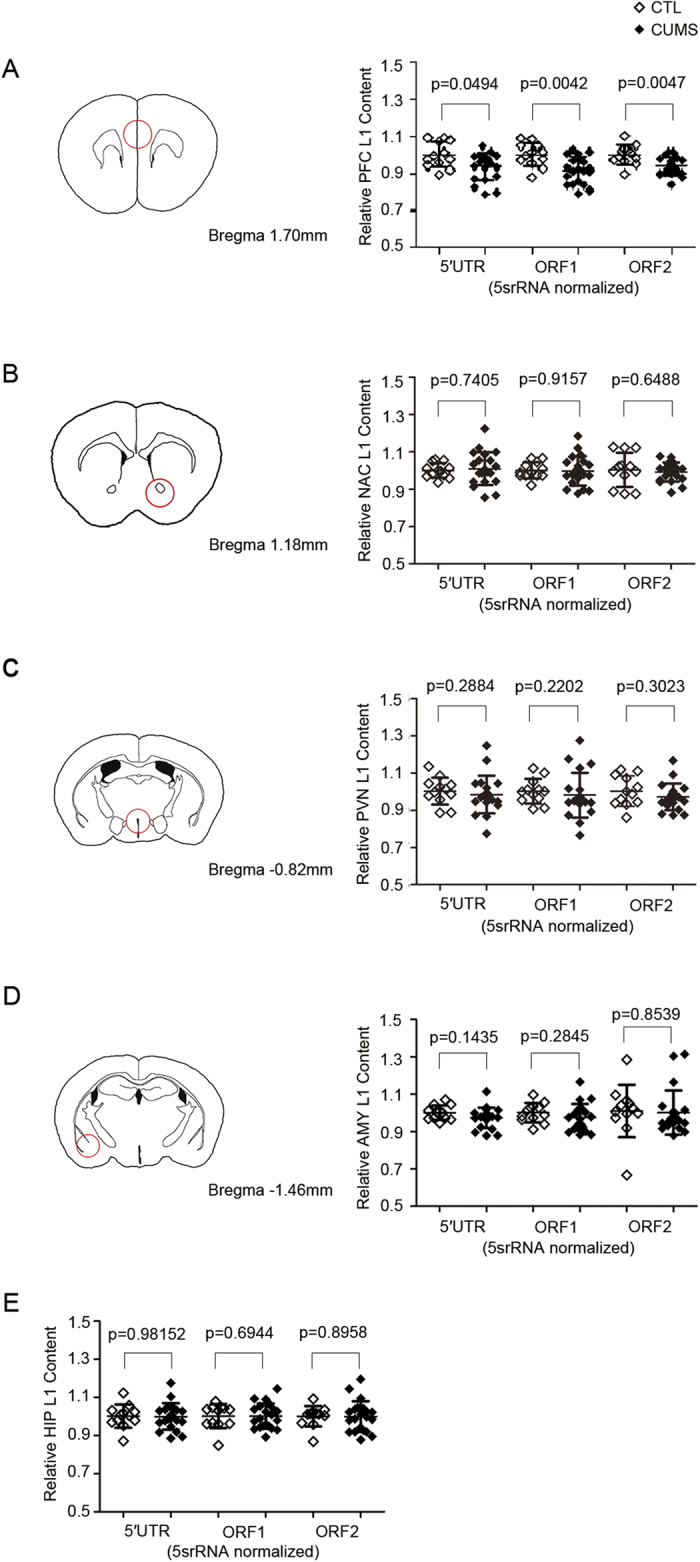
Decreased L1 copy number in the prefrontal cortex of CUMS mice. (**A)** Comparative Ct methods with 5s-rRNA as internal controls was used to quantify L1 content. In the prefrontal cortex, L1 copy number of stressed mice was significantly decreased (5′UTR, −6.3%, p = 0.0494; ORF1, −8.2%, p = 0.0042; ORF2, −6.0%, p = 0.0047). (**B**,**C**,**D**,**E)** Among other brain regions, there are no significant difference between controls and stressed mice. The Mann-Whitney U test was used to determine p values. Error bars indicate SD. CTL, controls (*n* = 12); CUMS, Chronic unpredictable mild stress (*n* = 21) except for PVN (n = 20). The schematic illustrations were drawn according to the sample slice and referred to the mouse brain atlas of Paxinos and Franklin (The Mouse Brain in Stereotaxic Coordinates, 2nd edition, San Diego, Academic Press, 2001)^60^.

It is well known that a long-term exposure to stress can trigger deleterious effects on the structure and function of the brain. Animals exposed to chronic stress have morphological and functional damages in various brain regions, including PFC and hippocampus^16^. In addition, there is mounting evidence that several subcortical structures related to motivation, reward and fear are also involved in depression, such as amygdala, NAC and hypothalamus. Thus, we selected PFC, hippocampus, amygdala, NAC and PVN to detect the changes of L1 copy number in our study, and found that L1 copy number was significantly decreased in PFC (5′UTR, −6.3%, p = 0.0494; ORF1, −8.2%, p = 0.0042; ORF2, −6.0%, p = 0.0047, Fig. 5A) in CUMS mice compared to controls. There was no significant difference between stressed mice and controls in the NAC, PVN, amygdala and hippocampus (p >* 0.05,* Fig. 5B,C,D and E), suggesting that decreased L1 copy number in PFC may also play an important role in the pathophysiology of MDD.

## Discussion

The occurrence of many disorders is related to genomic instability. L1 retrotransposition, as one of the DNA damaging agents, can lead to various types of genomic instability^17^,^18^. There is growing evidence that genomic instability can be caused by environmental stress^19^. Muotri *et al*. reported that voluntary exercise could increase L1 insertions in matured neurons in the hippocampus^20^. Some central nervous excitability drugs were found to influence L1 contents in the different brain regions^21^,^22^. The methylation of L1 is also easily affected by environments^23^,^24^. Genomic instability has been found to be associated with tumorigenesis, such as colon cancer and lung cancer^25^. In addition, previous studies have reported that genomic instability is a possible factor associated with many common neurological and psychiatric diseases^26^. L1 retrotransposition is commonly recognized to occur in germ cells, but recent studies reported ongoing retrotransposition in somatic cells, including stem cells, tumor cells, neural progenitor cells and early embryos cells^12^,^27^,^28^. The first example of somatic retrotransposition showed that L1 was inserted into the suppressor gene in colorectal cancer^29^. L1 retrotransposition mainly occurs in epithelial tumor such as prostate and ovarian cancers, but there has been no report on brain and blood cancers so far^25^. In addition to the role in cancers, L1 retrotransposition could produce somatic alterations in the brain. The first study about L1 retrotransposition in the central nervous system was an increase in L1 retrotranspositions in neuronal precursors from neural stem cells of rat hippocampus^30^. Furthermore, the experiments revealed that L1 element could retrotranspose in the hippocampus of human brain *in vivo*^12^,^20^. While L1 dysregulation may lead to various neurological disorders, whether L1 dysregulation directly causes neurological disorders remains unclear^12^,^13^,^31^,^32^,^33^. Increased L1 retrotransposon was discovered in Rett syndrome, a neurodevelopmental disorder^13^. Previous study also reported that L1 misregulation was involved in amyotrophic lateral sclerosis and frontotemporal lobar degeneration^4^. Li *et al*. revealed that the activation of L1 retrotransposition caused an age-dependent neuronal decline^34^. Furthermore, L1 dysregulation is also correlated with post-traumatic stress disorder (PTSD)^33^ and alcoholism^35^. Recently, a study discovered that schizophrenia patients had higher levels of L1 retrotransposition than controls and pointed out that L1 retrotransposition might contribute to both MDD and bipolar disorder^9^.

The present results suggested that the changes of L1 copy number might be related to MDD. We observed increased L1 copy number in peripheral blood of MDD patients and the animal model. We also found that L1 was robustly hypomethylated in peripheral blood of MDD patients compared to healthy controls. Possibly, increased L1 copy number in blood may be closely correlated with DNA demethylation in MDD. Hypomethylation in L1 may increase their activity, resulting in genomic instability and leading to many diseases, such as cancer^6^. L1 DNA hypomethylation has also been found in peripheral blood of schizophrenia patients^36^,^37^. Even though gene expression and methylation patterns in peripheral blood and brain may be different, the blood-based gene expression profiling has been applied to diagnose mental disorders in several studies^38^. Our method to measure the methylation levels of L1 5′-UTR is a straightforward and locus-specific DNA methylation assessment, which could measure the integral methylation levels in a selective region rather than methylation level at a single CpG site. Unfortunately, we failed to measure the methylation status of L1 in the mouse genome due to unavailability of technology in this study. Other methods such as pyrosequencing can be used to further confirm the finding in the future.

Furthermore, our results showed that L1 copy number was decreased in the prefrontal cortex of the animal model of depression. We speculate that there may be different mechanisms of regulating L1 retrotransposition between peripheral blood and the prefrontal cortex of depressive mice. Bundo *et al*. found that there was a tendency towards increase in L1 copy number in the prefrontal cortex of MDD patients. The differences in biological background of study samples may be the major reasons for the different results between our work and the study reported by Bundo *et al*. In addition, the extents of L1 changes were obviously smaller in CUMS mice than MDD patients in our study. This may be due to the small sample size in study of mouse model; mice exposed to CUMS for 35 days failed to completely express the symptoms observed in MDD patients.

There are several molecular mechanisms behind L1 retrotransposition repression. DNA methylation is the most common one to repress L1 retrotransposition^39^. In addition, there are some other mechanisms contributing to L1 repression. Yang *et al*. reported that small interfering RNAs could inhibit L1 retrotransposition at a post-transcriptional level through induction of mRNA degradation^40^. Both premature transcript termination and transcriptional elongation defects contributed to the repression of L1 retrotransposition^41^,^42^. Heras *et al*. proposed a model that microprocessor complex could bind to L1 mRNA and cleave hairpin structures, resulting in a decrease in L1 mRNA and retrotransposition rates ultimately^43^. Some proteins such as MOV10 and two members of the APOBEC3 family (APOBEC3A and APOBEC3B) can then inhibit L1 retrotransposition in cells^44^,^45^.

A number of brain regions have been reported to mediate the various symptoms of depression. Both human brain imaging and postmortem studies supported that depression involved structural and functional alterations in many brain regions including the hippocampus, PFC, amygdala, NAC and thalamus^16^. Postmortem studies failed to show atrophy of dendritic processes and decreased neuronal cell body size of limbic and cortical regions in depressed subjects^46^. Hippocampus and PFC are responsible for cognitive function, such as hopelessness and guilt^47^. Amygdala is the brain region to regulate emotional reactivity, which is involved in modulation of memory and motivation, anxiety and anhedonia^48^. NAC is regarded as a neural link between motivation and action, playing an important role in stress-related behavior, reward-motivated behavior and substance-dependence^49^. PVN is the final common neuronal pathway to regulate the hypothalamic-pituitary-adrenal (HPA) axis, providing an important hormonal component of stress responses^50^. Chronic stress can result in functional and morphological changes across these brain regions. For this reason, we established the model of chronic unpredictable mild stress to produce depressive-like features and explore the relationship between L1 and the limbic brain regions in depression. We found a decreased L1 copy number in PFC, but there was no change in other regions due to a mild CUMS given in our study. Previous studies demonstrated that there was the highest extent of changes induced by chronic stress in PFC^51^. Studies of post-mortem brain and imaging revealed that the volume of prefrontal cortex was decreased in MDD, consistent with a reduction in the size and density of neurons^52^,^53^. L1 mainly inserts in neurons of the central nervous system. Further study is needed to explore the mechanism by which L1 copy number is decreased either by stress directly or by the decreased number of neurons. The suppression of L1 activity may alter the expression of depression-related genes, such as those coding for dopamine receptor 3 (DRD3) and neurotransmitter transporters (SLC6A5, SLC6A6 and SLC6A9); L1 may also reshape the genetic circuitry in the neurobiological processes^30^,^54^. Several studies demonstrated that PFC was associated with the pathophysiology of depression^53^,^55^. These observations suggest that decreased L1 copy number in PFC may be triggered by environmental factors related to the pathophysiology of depression. In a word, the specific role of L1 in MDD should be further explored in the future.

To our knowledge, this is the first study to explore L1 copy number and its methylation status in peripheral blood of MDD patients. It is also the first time to investigate L1 copy number in five brain regions and in blood in the animal depression model. The results should be repeated in a larger sample of depression patients and animals, and additional animal models of depression is also needed to clarify the role of L1 in the MDD. In conclusion, L1 misregulation may be associated with MDD although L1 activity may be variable between peripheral blood and brain tissues. Possibly, there are different mechanisms that control L1 activity in both the central nervous system and peripheral blood.

## Methods

### Human subjects and animals

The complete details of the entire study design and procedures involved were in accordance with the Declaration of Helsinki. All patients and control subjects were of the Chinese Han origin from the northern area of China. They all gave written informed consent to attending this study as approved by the Ethics Committee of Chinese Academy of Medical Sciences and Peking Union Medical College. All animal work was approved by the Experimental Animal Center of Chinese Academy of Medical Sciences and Peking Union Medical College and in accordance with the institutional guidelines of the Beijing Administration Office of Laboratory Animals. This study was approved by the Institutional Review Board of Chinese Academy of Medical Sciences and Peking Union Medical College.

A total of 105 unrelated patients with MDD (45 males and 60 females, aged 43.3 ± 14.0 years) and 105 healthy controls (51 males and 54 females, aged 40.5 ± 3.5 years) were recruited for analysis of L1 copy numbers. Among them, 60 MDD patients (24 males and 36 females, aged 43.7 ± 14.7 years) and 41 healthy controls (19 males and 22 females, aged 39.7 ± 2.8 years) were used for analysis of L1 5′-UTR DNA methylation. All the patients were recruited as inpatients by First Hospital of Shanxi Medical University. Clinical diagnosis of MDD was made by at least two experienced psychiatrists according to the Diagnostic and Statistical Manual of Mental Disorders Fourth Edition (DSM-IV) criteria. Patients were interviewed with the same psychiatrists using the 17-item Hamilton Rating Scale for Depression (HAMD-17) during sampling. None of these patients received psychotropic medication within 4 weeks. Control subjects were recruited from local communities. The eligible controls were in physical health and did not have a history of psychiatric illness, serious somatic disease, alcoholism or drug abuse.

Male C57BL/6 J mice at 8–9 weeks of age (18–25 g; Vital River Animal Technology Co., Ltd., Beijing, China) were housed in groups of 2 per cage under a 12-h light/dark cycle at constant temperature (25 °C) with ad libitum access to food and water. All C57BL/6 J mice were allowed 1 week of habituation to the housing conditions before the start of experiments.

### Real-time quantitative PCR

QuickGene DNA whole blood kit (FUJIFILM, Cat. No. DB-L) was used with Automatic DNA/RNA Extraction System (FUJIFILM, QuickGene-610L, Japan) to isolate genomic DNA from human blood, which enables automatic extraction of about 50 μg of DNA from 2 ml of whole blood. Quantitative PCR experiments were performed using a CFX96 Real-Time PCR Detection System (Bio-Rad, Hercules, CA) according to standard protocol. The quantitative multiplexing PCR assay of human sample was performed using 100 pg DNA and SYBR-Green assay of mouse sample was performed using 500 pg DNA. Standard curves of genomic DNA ranging from 2.5 ng to 4 pg were performed with 5 times dilution. The slope of linear regression to the standard curve was nearly −3.32, which means that the primer efficiency and multiplexing effectiveness were acceptable. All RT-PCR reactions were performed in triplicates.

### Evaluation of L1 levels in blood

Human L1 copy number was measured with ORF2, HERVH or SATA as internal controls. The quantitative multiplexing PCR strategy to investigate endogenous L1 activity in humans was done as Gage *et al*. described^12^. ORF2 probes were conjugated to the fluorophore label HEX and HERVH/SATA probes were conjugated with 6FAM. A 20-μl reaction volume containing 10 μl of 2 × FastStart Essential DNA Probes Master Mix (Roche, Cat. No. 06924492001), 1 μl of primers F/R (5 umol/L), 1 μl of probe (2.5 umol/L) and 1 μl of DNA (100 pg/μl) was used for quantitative PCR amplification. The detailed quantitative PCR conditions were as follows: 95 °C for 10 min, followed by 36 cycles of amplification and quantification (95 °C for 10 sec, 58 °C for 20 sec and 72 °C for 20 sec).

### Evaluation of L1 5′-UTR DNA methylation in blood

Human L1 5′-UTR DNA methylation was measured by methylation sensitive restriction enzymes (MSREs) with quantitative RT-PCR (MSREs RT-PCR). The OneStep qMethyl™ kit (Zymo Research, Cat. No. D5310) was used to measure the level of DNA methylation. Primers used for L1 amplifications in 5′-UTR of human DNA sample were as follows: hL1-methF 5′-ACAGCTCCGGTCTACAGCTC-3′ and hL1-methR 5′-GTGTGGGATATAGTCTCGTGGTG-3′. The experimental procedure was based on manufacturer’s standard protocol. For each DNA sample to be analyzed, both a Test Reaction and a Reference Reaction mixture were set up properly. PCR was performed in a 20-μl reaction volume with 10 μl of 2 × Test Reaction PreMix or Reference Reaction PreMix, 5 μl of DNA (100 pg/μl) and 1 μl of mixed primers (5 μmol/L). The detailed quantitative PCR conditions were as follows: 37 °C MSREs digestion for 2 h, 95 °C for 10 min, followed by 45 cycles of amplification and quantification (95 °C for 30 sec, 60 °C for 1 min and 72 °C for 1 min), 72 °C final extension for 7 min.

The comparative Ct (2^−ΔΔCt^) method was employed to evaluate the relative quantity of L1 5′-UTR DNA methylation status. ΔCt = the Ct value from the Test Reaction minus the Ct values from the Reference Reaction. The large ΔCt represents the high status of non-methylated DNA.

### Chronic unpredictable mild stress procedure

The CUMS procedure was based on the model of Duman *et al*.^56^,^57^. In the CUMS model, animals were exposed to various kinds of mild stress, leading to anhedonia and learned helplessness, the two core symptoms of depression^58^. The CUMS animals were subjected to 11 stressors for 35 days (3 stressors per day from 1 to 21 days and 2 stressors per day from 22 to 35 days): hot stress, light on, light off, bedding deprivation, crowding, cold swimming, food and water deprivation, wet bedding, high platform, cage tilt, and restraint.

### Forced swimming test (FST)

On the day before the end of CUMS, mice were placed in a clear beaker with water (24 ± 1 °C) for 6 min. The procedure was recorded by video and the immobility time in the last 4 min was scored by an experienced observer who is blind to the mouse identities. Immobility was defined as floating or remaining motionless without leaning against the wall of the beaker^59^.

### Sucrose consumption test (SCT)

To determine whether mice acquired anhedonic phenotype induced by CUMS, a standard sucrose consumption assay was performed. Mice were habituated to drink 1% of sucrose solution (Sigma, St. Louis, MO) in a 50 ml conical tube for 48 h, followed by 12 h fluid deprivation. On the test day, mice were presented with 1% of sucrose solution for 1 h and the consumption was determined by the difference after weighing the conical tube pre- and post-test.

### DNA preparations from mouse blood and brain

After mice were deeply anesthetized with 10% chloral hydrate, 1 ml of eyeball blood was obtained and all mice were killed by cervical dislocation. Genomic DNA in blood was extracted by TIANamp Blood DNA Kit (TIANGEN, DP348) from 500 μl of whole blood. The brains were rapidly extracted and frozen in the cold isopentane for 30 sec, then stored at −80 °C until processed. Brains were embedded in the optimal cutting temperature compound (OCT, Leica) for 3 h at −22 °C in a cryostat (CM3050 S, Leica) to get equilibration of temperature before sampling. Tissues from different brain regions were sampled using a punch (Stoelting, Cat. No. 57401) with a diameter of 1.25 mm (Fig. 5). The hippocampus was directly dissected manually. Genomic DNA from punched tissues was extracted with a QIAamp DNA MicroKit (Qiagen, Cat. No. 56304) and DNA from the hippocampus was extracted with a QIAamp DNA Mini Kit (Qiagen, Cat. No. 51304).

### Evaluation of the L1 levels in blood and brain of CUMS

A 20-μl reaction volume containing 10 μl of 2 × FastStart Essential DNA Green Master Mix (Roche, Cat. No. 06924204001), 1 μl of mixed primers (8 μmol/L for 5srRNA and 2.5 μmol/L for others), and 1 μl of DNA (500 pg/μl) was used for quantitative RT-PCR amplification (5srRNA was used as an internal control). The detailed RT-PCR conditions were as follows: 95 °C for 10 min, followed by 36 cycles of amplification and quantification (95 °C for 10 sec, 61 °C for 1 min and 72 °C for 1 min). The blood and brains from twenty-one CUMS and twelve control mouse were quantified except that one sample of PVN did not meet our quality and was discarded.

### Statistical analysis

The ratio of L1 copy number was calculated and then normalized relative to the average value of control samples. The comparative Ct (2^−ΔΔCt^) method was used to quantify expression of L1 copy number and fold change (FC) was used to present data. Binary logistic regression was used to examine the difference in L1 levels and L1 5′-UTR DNA methylation between the patient group and control group with adjustment for age and sex. Pearson correlation was applied to analyze the correlation between the total score of HAMD-17 and either L1 levels or L1 5′-UTR DNA methylation. Mann-Whitney U test was used to examine the differences in L1 copy number, sucrose consumption and immobility time between CUMS mice and controls.

## Additional Information

**How to cite this article**: Liu, S. *et al*. Inverse changes in L1 retrotransposons between blood and brain in major depressive disorder. *Sci. Rep.* **6**, 37530; doi: 10.1038/srep37530 (2016).

**Publisher’s note:** Springer Nature remains neutral with regard to jurisdictional claims in published maps and institutional affiliations.
